# The influence of tamoxifen on normal mouse mammary gland homeostasis

**DOI:** 10.1186/s13058-014-0411-0

**Published:** 2014-07-24

**Authors:** Mona Shehata, Renée van Amerongen, Amber L Zeeman, Rajshekhar R Giraddi, John Stingl

**Affiliations:** 1 Cancer Research UK Cambridge Institute, University of Cambridge, Li Ka Shing Centre, Robinson Way, Cambridge, CB2 0RE UK; 2 Section of Molecular Cytology, Swammerdam Institute for Life Sciences, University of Amsterdam, Science Park 904, 1098 XH Amsterdam, Netherlands

## Abstract

**Introduction:**

Lineage tracing using inducible genetic labeling has emerged to be a powerful method for interrogating the developmental fate of cells in intact tissues. A common induction mechanism is the use of tamoxifen-dependent Cre recombinase (CreER and CreER^T2^), but the effects of tamoxifen at doses normally used in lineage-tracing studies on normal adult mammary gland homeostasis are not known.

**Methods:**

We used flow cytometry and immunostaining of intact glands to determine whether varying doses of tamoxifen skew the distribution and the apoptosis and proliferation status of different types of mammary epithelial cells *in vivo*. We also examined how tamoxifen influences the number of progenitor and mammary repopulating units (MRUs).

**Results:**

Our results indicate that ≥5 mg/25 g body weight of tamoxifen induces a transient increase in cell proliferation and in the number of basal cells in the adult mammary epithelium up to 7 days after tamoxifen administration. However, in the medium term (3 weeks), all doses of tamoxifen ≥1 mg/25 g body weight result in a decrease in the number of basal and EpCAM^+^CD49b^−^ luminal cells and a decrease in progenitor cell function. Tamoxifen at doses ≥5 mg/25 g body weight induced a transient increase in caspase-3-mediated apoptotic cell death within the mammary epithelium. However, mammary epithelial cell numbers in all subpopulations were restored to their original levels by 8 weeks. No long-lasting effects of tamoxifen on MRU numbers or on pubertal ductal development were observed.

**Conclusion:**

Tamoxifen can skew the distribution of mammary cell types in a dose-dependent manner, and thus caution must be taken when interpreting lineage-tracing studies using high doses of tamoxifen, particularly when short-duration analyses of a quantitative nature are being performed.

**Electronic supplementary material:**

The online version of this article (doi:10.1186/s13058-014-0411-0) contains supplementary material, which is available to authorized users.

## Introduction

Lineage tracing has emerged to become a powerful method for establishing hierarchical relations between stem and progenitor cells within epithelial tissues [1]. The main advantage of lineage-tracing strategies is that they permit the interrogation of the growth and differentiation potentials of distinct subsets of cells in a homeostatic-tissue context, thus bypassing the artefacts associated with tissue dissociation and transplantation assays [2]–[4].

One common lineage-tracing approach involves the use of a specific promoter to direct expression of Cre recombinase to defined subsets of cells, such that these cells and their progeny are irreversibly marked with a reporter gene. By using an inducible system, the timing and the proportion of cells that undergo genetic recombination can be refined, such that the contribution of specific cells to tissues at defined developmental time points can be interrogated at clonal densities. One common mechanism includes the tamoxifen-inducible Cre recombinase CreER, and the more refined CreER^T2^[5]–[7].

One assumption of lineage tracing is that the inducing agent itself (that is, tamoxifen) does not affect homeostasis of the tissue under study. However, reports have demonstrated that tamoxifen, at doses that are commonly used to induce lineage tracing, can induce apoptosis within the gastrointestinal tract and, and in some cases, can influence the outcome of lineage-tracing experiments [8],[9].

Considering that tamoxifen is an estrogen-receptor (ER) antagonist and is a commonly used drug in the treatment of breast cancer to inhibit the proliferation of estrogen receptor^+^ (ER^+^) breast cancer cells [10], we investigated the effects of a range of doses of tamoxifen on the distribution and cell-division kinetics of the different mammary cell populations in the intact adult mouse mammary gland. We also examined the influence of tamoxifen on pubertal mammary gland development.

Our results indicate that tamoxifen, at relatively high doses, induces a transient increase in cell proliferation and basal cell numbers in the mammary epithelium. In the longer term (3 weeks), all doses of tamoxifen ≥1 mg/25 g mouse result in decreased numbers of basal and EpCAM^+^CD49b^−^ luminal cells and decreased progenitor cell function, although these populations recover by 8 weeks.

## Methods

### Mice and tamoxifen treatment

To determine the effects of tamoxifen on outgrowth of the mammary epithelium during puberty, 23-day-old C57BL/6 J mice received a single intraperitoneal (IP) injection of sunflower oil, 1 mg of tamoxifen in oil/25 g body weight, or 4 mg of tamoxifen in oil per 25 g body weight. Mammary glands were harvested on postnatal day 43 and stained with carmine alum for whole-mount analysis.

To determine the effects of tamoxifen on the mammary glands of adult mice, 10– to 14-week-old C57BL/6 J mice were given a single IP injection of 0.2 mg, 1 mg, or 5 mg tamoxifen (dissolved in 100% corn oil), or three IP injections of 5 mg every other day. Inguinal mammary glands were then resected from the mice 1 to 56 days after the last tamoxifen injection. The resected glands were then dissociated and processed to a single-cell suspension, as previously described [11]. Mice used for phenotypic analysis and colony-forming cell (CFC) assays were estrus staged [12],[13] immediately before culling and tissue harvest. All mice were obtained from Charles River (Hertfordshire, UK) and housed in open cages and provided with food and water *ad libitum* at the laboratory animal facility at the Netherlands Cancer Institute and at the Cambridge Institute. All experiments were conducted in accordance with institutional guidelines and national regulations, and all experimental procedures were approved by the Institutional Animal Care and Use Committee (DEC) of the Netherlands Cancer Institute and by the Cambridge Institute Animal Ethics Committee.

### Whole-mount carmine staining

The thoracic and inguinal mammary glands were flat fixed for 4 hours in a 1:1 mixture of ethanol and acetic acid. After fixation, the glands were washed with 70% ethanol for 1 hour, rinsed in water, and stained overnight in carmine alum staining solution. Stained glands were washed in 100% ethanol and cleared in orange terpene (Histoclear, National Diagnostics). All steps were carried out at room temperature.

### Measuring epithelial outgrowth

Carmine-stained mammary glands were photographed by using a Leica MZFLIII stereomicroscope equipped with a Nikon DXM1200 digital camera. Relative growth of the mammary epithelium was scored in the inguinal glands by drawing a tangent line, perpendicular to the distal edge of the lymph node. A second line was drawn parallel to the tangent at the most distal tip of the epithelium. The distance between these parallel lines was scored as “distance from the lymph node”.

### Flow cytometry

Mouse mammary cells were preblocked with 10% normal rat serum and then incubated with the following primary antibodies: CD31-biotin (clone 390, eBioscience), CD45-biotin (clone 30-F11, eBioscience), Ter119-biotin (clone Ter119, eBioscience), BP-1-biotin (clone 6C3, eBioscience), EpCAM-APC (clone G8.8, BioLegend), CD49f-AF488 (clone GoH3, BioLegend), CD49b-PE (HMα2, BioLegend), and Sca1-PE/Cy7 (clone D7, BioLegend). In some experiments, cells were stained with CD24-PE (clone M1/69, BioLegend) and CD29 (clone HMβ1-1, BioLegend). CD45, Ter119, CD31, and BP-1 were used to deplete contaminating hematopoietic, endothelial, and a proportion of stromal cells, respectively (collectively termed Lin + cells). Biotin-conjugated antibodies were detected with Streptavidin-APC-Cy7 (BioLegend). Cells were then filtered through a 30-μm cell strainer and incubated with 4′,6-diamidino-2-phenylindole (DAPI; Invitrogen) and were analyzed by using an LSRII (Becton Dickinson) and were sorted on a FACSAria I (Becton Dickinson). The flow-cytometry gating strategy was as previously described [11].

### Mammary repopulating unit assay and *in vitro*CFC assays

For the MRU assays, donor cells were suspended in 65% Hanks Balanced Salt Solution supplemented with 2% fetal bovine serum (FBS), 25% Growth Factor Reduced Matrigel (Becton Dickinson), and 10% Trypan Blue solution (0.4%, Sigma) at a concentration such that a 10-μl injection volume contained the desired cell dose. The endogenous mammary epithelium in the inguinal glands of 3-week-old female C57BL/6 J mice was cleared, and cells were injected into cleared fat pads, as previously described [14]. The mice were mated 3 weeks after surgery, and the inguinal glands were removed during pregnancy and glands fixed in Carnoy fixative and stained with carmine alum. An outgrowth was scored positive if it contained both lobular and ductal elements. MRU frequencies were calculated by using the Extreme Limiting Dilution Analysis (ELDA; [15]) tool.

The CFC assays were performed as previously described [11],[16]. In brief, cell suspensions of 1,000 sorted mouse luminal mammary cells were seeded in Mouse EpiCult-B (StemCell Technologies) and 50 μg/ml gentamicin in the presence of irradiated feeders for 6 to 7 days. In some experiments in which 1,000 basal cells were seeded, FAD media (3:1 DMEM/F12 (+1.8 × 10-4 *M* adenine + 1.8 × 10-3 *M* calcium) + 10% FBS (PAA) + 0.5 μg/ml hydrocortisone (Sigma) + 10-10 *M* cholera toxin (Enzo Life Sciences) + 10 ng/ml epidermal growth factor (EGF, Peprotech) + 5 μg/ml insulin + 10 μ*M* Y-27632 (Sigma) + 50 μg/ml gentamicin) was used instead of Mouse EpiCult-B.

At the end of the assays, the colonies were fixed with acetone/methanol (1:1), stained with Giemsa (Fisher Scientific), and enumerated under a microscope. CFCs per pair of inguinal glands was calculated by multiplying the cloning efficiency by the subpopulation cell number.

### Immunofluorescence and immunohistochemistry

Paraffin-embedded mammary tissues were sectioned at 4 μm, deparaffinized, and boiled in pH 6.0 citrate buffer. For immunofluorescence, sections were blocked in 1% BSA/0.1% Tween-20/PBS for 1 hour and incubated with primary antibodies specific for keratin 5 (Abcam), ER (Novocastra), and Ki-67 (Dako) overnight at 4°C. Goat anti-mouse, anti-rabbit, and anti-rat antibody conjugated to Alexa Fluor (AF)555, AF647, and AF488, respectively, were used to detect primary antibodies. No primary antibody was used as a control. Nuclei were visualized with DAPI, and sections were mounted with ProLong Gold Antifade (Invitrogen). Slides were scanned on the Leica Ariol imaging system by using an Olympus BX61 microscope (Leica).

Immunohistochemistry staining for Ki-67 (Dako) and CC3 (Vector Labs) was performed on an automated BondMax (Leica). Slides were scanned by using the Scan ScopeXT Imaging System (Aperio).

### Statistical analysis

**Figure 1:**
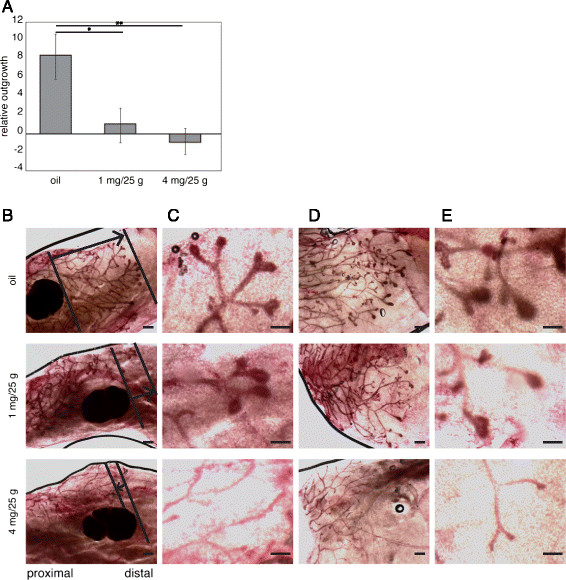
**Tamoxifen temporarily retards ductal elongation during pubertal mammary gland development. (A)** Bar graph depicting the quantification of mammary gland outgrowth, showing relative elongation of the inguinal mammary gland between P23 and P43 in mice treated with sunflower oil (*n* = 4), 1 mg/25 g of tamoxifen (*n* = 4), or 4 mg/25 g of tamoxifen (*n* = 4). The position of the most distal tip of the lymph node is set at zero. (*P < 0.05, **P < 0.01). **(B)** Whole-mount preparations of carmine alum-stained mammary epithelium, showing representative images of the inguinal glands quantified in panel (A). Arrows indicate the relative outgrowth of the epithelium. Scale bar = 500 μm. **(C)** Close-up of the glands depicted in (B), showing loss of terminal-end bud structures in mice treated with 4 mg/25 g tamoxifen. Scale bar = 20 μm. **(D)** Whole-mount carmine alum staining of thoracic glands. Scale bar = 500 μm. **(E)** Close-up of thoracic glands depicted in (D). Scale bar = 20 μm.

Data presented are the means of multiple independent mice (*n* = 4 to11 for all experiments except for the 0.2-mg dose, for which *n* = 3–4) and the standard error of the mean, except in Figure 1, where the standard deviation of the mean is shown. Two-way analysis of variance was used to test multiple groups followed by Dunnett multiple comparisons test to test significant differences between results. Significance was set at *P < 0.05, **P < 0.01, or ***P < 0.0001.

A Fisher Exact test was performed on 10 contingency tables measuring tamoxifen dosage against estrus stage in mice over a series of days. The results were corrected for multiple testing by using a Bonferroni correction. Significance was set at *P* < 0.05.

A Student *t* test was performed on the data depicted in Figure 1. Significance was set at * *P* < 0.05 and **P < 0. 01.

## Results

### Tamoxifen inhibits outgrowth of the mammary epithelium during puberty

Tamoxifen-mediated Cre recombination has been used successfully to label cells in the mammary gland at different developmental time points, but the dose of tamoxifen used to achieve sufficient labeling varies between studies (Additional file 1: Table S1).

To determine the effects of tamoxifen on ductal development, we injected wild-type C57BL/6 J mice with either a low (1 mg/25 g) or a high (4 mg/25 g) dose of tamoxifen at the onset of puberty. Next, we measured outgrowth of the mammary epithelium approximately 3 weeks later and compared the extent of growth from tamoxifen-treated mice with that of mice that had received an injection only with sunflower oil. Our measurements show that even a low dose of tamoxifen (1 mg/25 g), administered during puberty, significantly delays mammary gland development (Figure 1A). A similar reduction in pubertal ductal development has been reported when tamoxifen is administered to neonatal mice [17]. In addition, high doses of tamoxifen result in an apparent collapse of terminal-end bud structures in both the inguinal and the thoracic gland (Figure 1B-E). Despite this inhibition, ductal development ultimately catches up, and complete ductal outgrowth was eventually obtained in adult mice [4], which is consistent with a previous report [18].

We next set out to test the immediate and long-term effects of different doses of tamoxifen on the different mammary cell types in adult mice.

### Tamoxifen skews the distribution of epithelial cells in the mammary gland

**Figure 2:**
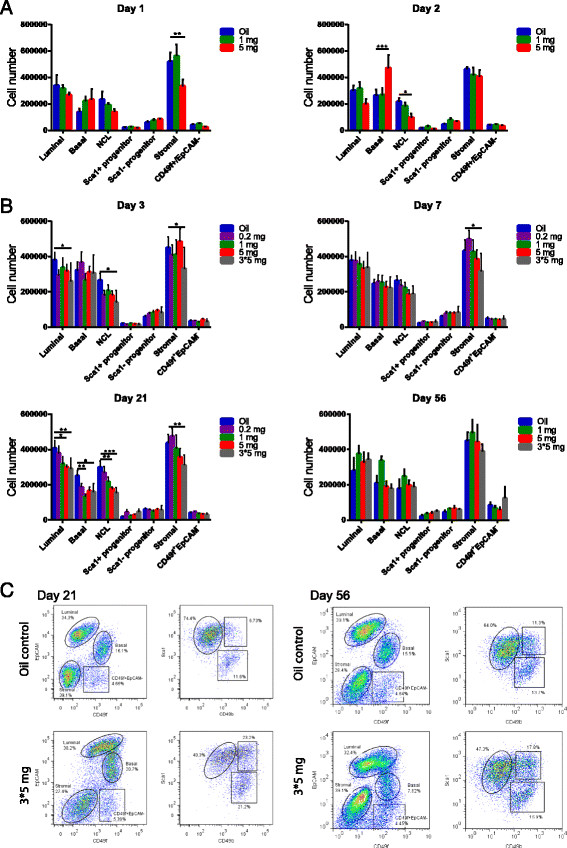
**High doses of tamoxifen can skew the relative distribution of mammary cell populations over time.** Absolute number of different types of mammary cells per pair of inguinal glands treated with varying doses of tamoxifen and analyzed at **(A)** 1 and 2 or **(B)** 3, 7, 21, and 56 days after tamoxifen injection (*P < 0.05; **P < 0.01; ***P < 0.0001). **(C)** Representative flow-cytometry dot plots show the expression of EpCAM, CD49f, Sca1, and CD49b among mammary cells from control mice and mice treated with 3 × 5 mg tamoxifen and analyzed 21 (left panels) or 56 days (right panels) later.

Mouse mammary epithelial cell populations can be resolved by flow cytometry on the basis of differential expression of EpCAM, CD49f, Sca1, and CD49b (Figure 2C, top panels). Basal cells have an EpCAM^low^CD49f^high^ phenotype, whereas undifferentiated and differentiated luminal progenitors have an Sca1^−^CD49b^+^ and an Sca1^+^CD49b^+^ phenotype, respectively. The most-differentiated cell population, the non-clonogenic luminal (NCL) cells, have an Sca1^+^CD49b^−^ phenotype [11].

To investigate the influence of tamoxifen on these different cell populations, adult female mice were injected with either corn oil or with 1 mg or 5 mg of tamoxifen. Mice were culled 1 and 2 days later, and the absolute number of cells of different phenotypes per pair of inguinal glands was enumerated by cell counting and flow-cytometry analysis. We observed that by day 2, the 5-mg dose of tamoxifen caused a slight decrease in the number of luminal cells present in the mammary gland (Figure 2A). Unexpectedly, we also observed a 56% increase in the number of basal cells within the mammary epithelium compared with oil control.

We then repeated these experiments by using a broader range of tamoxifen doses (0.2 mg, 1 mg, 5 mg, or 3 × 5 mg) and analyzed the mice at 3, 7, 21, and 56 days after the last injection. We observed that the previously observed increase in basal cell number was only transient, because it was no longer detected after 3 or more days. In mice injected with ≥1 mg of tamoxifen and analyzed 21 days later, the absolute number of both basal and NCL cells had decreased by 61%, 40%, and 44% and 32%, 51%, and 64%, in 1 mg, 5 mg, and 3 × 5 mg, respectively, although the two luminal progenitor populations remained unchanged (Figure 2B). We also observed a slight decrease in the stromal population at the highest tamoxifen dose, and this may be because the stromal compartment, like many of the luminal epithelial cells, also expresses ER [19]. When the experiment was extended to 56 days, the number of basal and NCL cells recovered to that of the oil control for all doses (Figure 2B). The proportions of these subpopulations relative to each other in control and tamoxifen-treated mice is shown in Additional file 2: Figure S1.

We observed that the phenotypes of these cells changed after administrating the higher doses (5 and 3 × 5 mg) of tamoxifen. The luminal and basal cell populations, which are normally resolved as two distinct subpopulations on a dot plot, began to merge with one another in tamoxifen-treated mice (Figure 2C, lower panels, and see Additional file 3: Figure S2). When the luminal subpopulations were back-gated, we observe that this gain in CD49f expression was mostly restricted to the Sca1^+^ progenitors and the NCL cells (Additional file 4: Figure S3A). Examination of the effects of tamoxifen on the expression of the mammary epithelial markers CD24 and CD29 revealed that these markers are not influenced by tamoxifen, as no discernable differences in the dot plots were observed between control mice and mice receiving high doses of tamoxifen (Additional file 4: Figure S3B).

### Higher doses of tamoxifen decrease the number of basal CFCs but not MRUs in mammary glands

**Figure 3:**
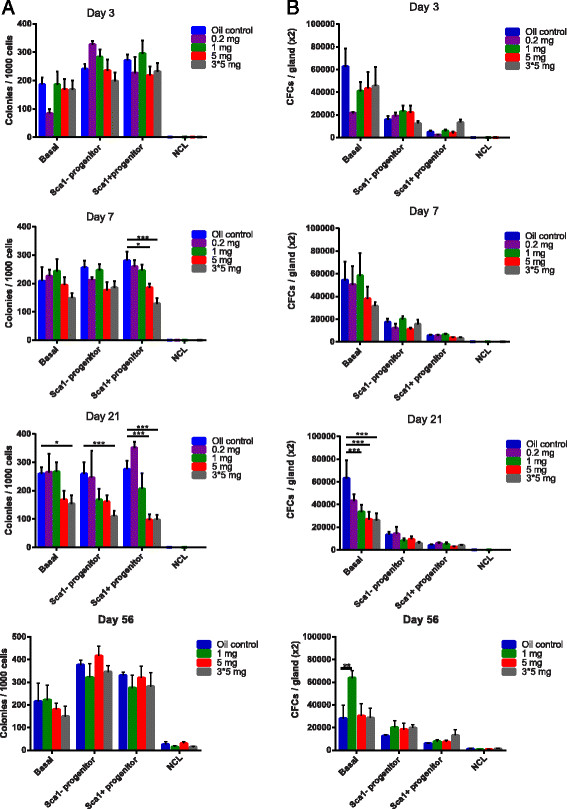
**Tamoxifen can decrease the number of CFCs in the mammary epithelium. (A)** Cloning efficiency and **(B)** total number of CFCs per pair of inguinal mammary glands in mice treated with varying doses of tamoxifen and analyzed at 3, 7, and 21 days after treatment. (*P < 0.05; **P < 0.01; ***P < 0.0001); *n* = 5 to 11 for all experiments, except for 0.2-mg dose, where *n* = 3 to 4.

When we examined the influence of tamoxifen on the cloning efficiency of purified subpopulations of epithelial cells, we observed that, in general, only the higher doses (5 mg and 3 × 5 mg) influenced the cloning efficiencies (Figures 3A and Additional file 5: Figure S4). This effect was seen for the basal cells, Sca1^−^ luminal progenitors, and the Sca1^+^ luminal progenitors, but only at 7 and 21 days after tamoxifen administration. We also observed that a 1-mg dose of tamoxifen did result in a slight decrease in the cloning efficiency of Sca1^−^ luminal progenitors per 1,000 cells plated and detected 21 days later (Figure 3A). However, when we corrected this for total population size by multiplying the cloning efficiency with cell number, we observed that the total number of luminal CFCs per pair of inguinal glands did not discernibly change with tamoxifen treatment. However, tamoxifen did have a profound effect on the basal compartment, where even doses of 1 mg tamoxifen significantly decreased the absolute number of basal progenitors present in the glands at 21 days after treatment (Figure 3B).

We interrogated the effects of tamoxifen on the number of MRUs present in the mammary gland. We observed that mammary glands of mice treated with 1 mg and 5 mg of tamoxifen had no statistical differences in the frequency of MRUs when compared with oil-treated controls when examined 3 days (1 mg = 1 MRU in 1,850 cells; 5 mg = 1 MRU in 361 cells) and 21 days (1 mg = 1 MRU in 326 cells; 5 mg = 1 MRU in 467 cells) after tamoxifen injection (Additional file 6: Table S2).

One unusual observation that we noted during the course of these experiments is that mice treated with higher doses of tamoxifen tended to have an increased probability of being in metestrus than in other stages of the estrus cycle (Additional file 7: Table S3). Although statistical significance was not obtained for this observation, two different time points (Day 3 and Day 7) demonstrate a trend for this phenomenon (*P* = 0.06 and 0.07, respectively). This effect is transient, and mice examined at the Day 21 time point did not exhibit this behavior.

### Tamoxifen induces short-term cell proliferation and cell death in the mammary epithelium

**Figure 4:**
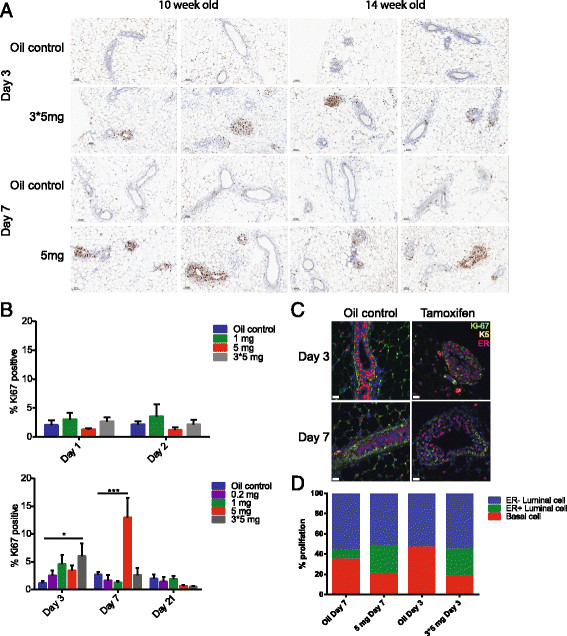
**Tamoxifen can induce short-term proliferation of mammary epithelial cells. (A)** Ki-67-immunostained sections of mammary glands from oil- and tamoxifen-treated mice at selected time points for 10–week- and 14-week-old treated mice. Scale bars = 50 μm. **(B)** Frequency of Ki-67^+^ cells in mammary glands of mice treated with oil or varying doses of tamoxifen, and analyzed at multiple time points. **(C)** Representative immunofluorescent sections of mammary glands from oil- and tamoxifen-treated glands at time points Days 3 and 7. Shown is the expression of keratin 5 (yellow), ER (red), and Ki-67 (green). Nuclei were visualized with DAPI. Scale bar = 25 μm. **(D)** Proportion of proliferating ER^−^ luminal cells, ER^+^ luminal cells, and basal cells in tamoxifen-treated glands. (*P < 0.05; **P < 0.01; ***P < 0.0001).

Given the change in basal and luminal cell numbers after tamoxifen application, we next examined the effect on proliferation of mammary epithelial cells. We observed an increase of Ki-67^+^ cells and the luminal cells forming a thick pluristratified epithelium in mammary glands of mice that received the highest doses of tamoxifen (Figure 4A). To ensure that this observation of increased Ki-67^+^ in mammary epithelial cells is due to tamoxifen administration and not a delay in puberty, we repeated this experiment by using 14-week-old mice and observed the same phenomenon (Figure 4A). This increase in proliferation is evident at the Day 3 time point for the 3 × 5-mg tamoxifen dose and at the Day 7 time point for the 5-mg dose (Figure 4B). Because the highest dose (3 × 5 mg) is administrated over several days, this results in a total of 7 days after the first 5-mg injection, and thus the effect is similar to the result observed with the 5-mg dose. At high tamoxifen doses (5 mg or 3 × 5 mg), tamoxifen increases the proportion of proliferating ER^+^ cells and decreases the proportion of proliferating basal cells compared with oil controls (Figure 4C, D).

**Figure 5:**
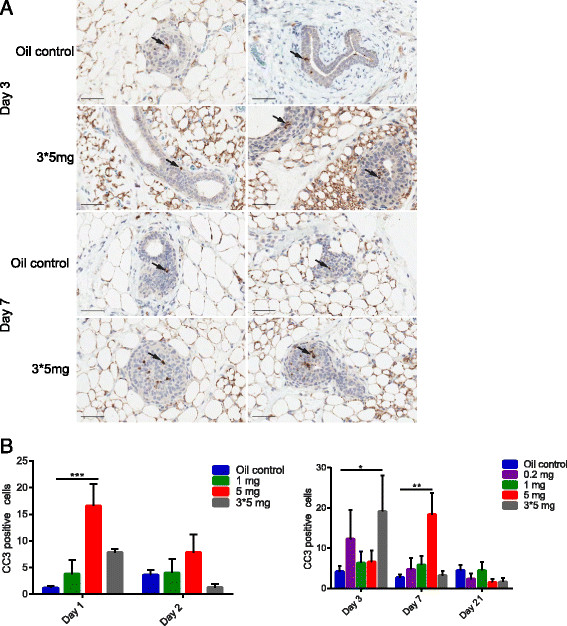
**Tamoxifen can induce slight short-term apoptotic activity in mammary epithelial cells. (A)** CC3 immunostained sections of mammary glands from oil- and tamoxifen-treated mice at selected time points. Arrows indicate CC3-positive cells. **(B)** Bar charts depicting the number of CC3^+^ cells per pair of oil- and tamoxifen-treated glands analyzed at 1, 2, 3, 7, and 21 days after treatment. (*P < 0.05; **P < 0.01; ***P < 0.0001).

To assess whether this transient increase in cell proliferation is accompanied by increased apoptosis, we also immunostained mammary tissue sections to detect cleaved caspase-3 (CC3), one of the key executioners of apoptosis. We observed a biphasic increase in the number of CC3^+^ cells in mammary glands of mice that received a 5-mg tamoxifen dose, with peaks on Day 1 and Day 7. A peak in apoptotic cells on 3 days after the last injection was observed when mice were administered three successive 5-mg doses spread over 5 days, which correlates to 7 days after the first dose. Although tamoxifen could increase the frequency of CC3^+^ within the mammary glands, the overall frequency of apoptotic cells in both oil- and all tamoxifen-treated mice is exceedingly low (Figure 5A, B). The frequency of this tamoxifen-induced apoptosis is likely an underestimate of the true frequency, because it would be expected that resident macrophages would quickly clear the dying cells. As well, the presence of CC3^+^ cells at any one point of time would likely not be representative of the total amount of apoptosis if cell death were nonsynchronous within the epithelial cell population.

Similar patterns exist between increased proliferation and cell death, whereby increased numbers of CC3 cells can be detected up to 7 days after initial 5-mg or 3 × 5-mg tamoxifen injections (Figure 5B). By 21 days after tamoxifen administration, a trend in the number of both proliferating and dying cells in the higher doses, although this is not significant (Figures 4B and 5B). Taken together, this indicates that tamoxifen is stimulating proliferation as well as a low degree of apoptosis within a short period after tamoxifen administration.

## Discussion

Inducible lineage tracing has rapidly become the new gold standard in assessing cell fate and identifying stem cells in intact tissues [1],[2],[4],[20],[21]. Most lineage-tracing models described to date rely on the tamoxifen-induced CreERT^2^-mediated recombination. The doses commonly used by researchers to obtain recombination in the mammary epithelium tend to be higher than those used for other organs, and the concern arises that these high doses of tamoxifen may have unrecognized off-target effects that may influence the fate of the cells under study. The results presented here show that tamoxifen, at least during the first 3 weeks after administration, decreases overall mammary epithelial cellularity in a dose-dependent manner, with NCL and basal cells being particularly affected, although this cellularity is restored by 8 weeks after injection. Surprisingly, we observed that higher doses (for example, 5 mg) of tamoxifen caused a transient short-term increase in the number of basal and proliferating cells in the epithelium. The mechanism behind this is not clear, but we hypothesize that this is mediated by increased levels of circulating female sex steroid hormones, because previous studies in humans demonstrated that tamoxifen can transiently increase estrogen production from the ovaries [22]–[24], and a study in mice demonstrated that neonatal exposure to tamoxifen can increase circulating progesterone levels [17].

Related to this, we also observed that tamoxifen can induce transient skewing of the estrus cycle, because mice treated with high doses of tamoxifen tend to have an increased probability of being staged in metestrus. The mechanism responsible for this phenomenon is not known.

We observed that tamoxifen induces a transient delay in pubertal ductal development. A similar observation was recently reported by the Visvader laboratory [25]. This is not surprising, considering that tamoxifen is a cytostatic compound that causes cells to remain in early G1 phase of the cell cycle [26],[27]. We hypothesize that this delay in development is the result of the time it takes for tamoxifen to be cleared from the mammary tissue, because it was previously reported that tamoxifen and its metabolites are detectable in mice 7 days after administration of doses (for example, 5 mg/25 g) comparable to those described here [28].

We report that high doses of tamoxifen (for example, 3 × 5 mg) cause the NCL cells to increase their expression of CD49f. A similar dose-dependent effect of tamoxifen on *Itga6* transcription was previously reported for uterine cells [29]. *ITGA6*, the gene that encodes CD49f, has been previously reported to be regulated by ERα [30], and the data presented here suggest that tamoxifen has similar agonist activities on *Itga6* transcription. We did not observe a dose-dependent effect of tamoxifen on CD29, and we would suggest that this marker would be superior to CD49f for resolving mammary epithelial cell populations from tamoxifen-treated mice with flow cytometry.

It is not known whether these off-target effects of tamoxifen influence the fate of the cells in lineage-tracing experiments in the mammary gland. However, lineage-tracing experiments using doxycycline-inducible K14-rtTA/TetO-Cre mice have yielded results similar to those in tamoxifen-inducible K5-CreER mice [2]. Because K14 and K5 have similar, if not identical distributions in the mouse mammary epithelium, this suggests that tamoxifen does not adversely affect the fate of the cells in these experiments.

It was demonstrated that in the intestine, tamoxifen reduces the efficiency of lineage tracing from Lgr5^+^ cells. Moreover, it induces apoptosis of the Bmi-1^+^ cells in the intestinal crypt, and the stem cell properties of these cells are greatly reduced unless apoptosis is suppressed [9]. Tamoxifen is known to induce apoptosis by multiple mechanisms in mammary tumor cells [31], including caspase-3-mediated apoptosis [32]. We observed an induction of caspase-3-mediated apoptosis in the mammary epithelium on administration of doses of tamoxifen >5 mg. This response appears to be biphasic, with a peak in apoptosis 1 day and 7 days after tamoxifen administration. It previously was reported that progesterone can induce a biphasic proliferation response in the mouse mammary epithelium, with an early immediate direct response and a delayed second wave via paracrine factors [33]. We hypothesize that an opposite, but analogous biphasic response is occurring with tamoxifen treatment; that is, tamoxifen induces immediate apoptosis in some cell types (the first apoptotic wave), and then through loss of paracrine interactions, a second wave several days later. However, it is important to note that the overall levels of CC3-mediated apoptotic cell death were still very low when compared with the total epithelial cell population at all tamoxifen doses tested. Whether the off-target effects of tamoxifen can influence the results of a lineage-tracing experiment will ultimately depend on the gene promoter being used to direct the trace, the dose of tamoxifen used, and the duration of the experiment. However, our results demonstrate that caution and, where possible, controls must be taken when interpreting short-duration lineage-tracing studies that are of a quantitative nature, especially when using high doses of tamoxifen.

## Conclusion

The advantage of inducible lineage tracing is that it permits the interrogation of the fate of cells at a clonal level in intact tissues, with the assumption that the lineage trace itself does not influence the homeostatic state of the tissue. Data presented here demonstrate that a single short-term exposure of tamoxifen at doses that are commonly used in mammary lineage-tracing studies can transiently increase basal cell numbers and cell proliferation within the mammary epithelium, although in the midterm (~3 weeks), a general decrease is found in the cellularity of the epithelium. However, by 8 weeks, all epithelial cell populations are restored to their original levels.

Higher doses of tamoxifen can also induce a transient increase in caspase-3-mediated apoptosis and transient disruptions in normal estrus cycling. No long-lasting effects of tamoxifen on MRU numbers are observed. Although these results do not diminish the power and utility of inducible lineage-tracing studies, they do indicate that potential off-target effects should be considered during the design and analysis of these experiments.

## Additional files

## Electronic supplementary material

Additional file 1: Table S1.: Summary of lineage-tracing studies in the mammary epithelium. (PDF 8 KB)

Additional file 2: Figure S1.: Distribution of mammary cell types in mice injected with oil or with varying doses of tamoxifen and analyzed 3 to 56 days later. (PDF 2 MB)

Additional file 3: Figure S2.: Gating of mouse mammary epithelial and luminal subpopulations of different doses analyzed at various time points. (PDF 3 MB)

Additional file 4: Figure S3.: (A) Back-gating of NCL, Sca1^+^, and Sca1^−^ progenitors from various doses analyzed 21 days after tamoxifen administration. Solid black line, CD49f expression levels in oil-treated control mice. (B) Comparing mouse epithelial subpopulations with CD24/CD29 or EpCAM/CD49f at Day 21. (PDF 1 MB)

Additional file 5: Figure S4.: CFC plates from a representative experiment 21 days after treatment. (PDF 460 KB)

Additional file 6: Table S2.: Frequency and absolute number of MRUs in oil- and tamoxifen-treated mice. (PDF 13 KB)

Additional file 7: Table S3.: Distribution of mice in different stages of the estrus cycle after tamoxifen treatment. (PDF 13 KB)

Below are the links to the authors’ original submitted files for images.

Authors’ original file for figure 1

Authors’ original file for figure 2

Authors’ original file for figure 3

Authors’ original file for figure 4

Authors’ original file for figure 5

## Abbreviations

AF: Alexa Fluor
CC3: cleaved caspase 3
CFC: colony-forming cell
ER: estrogen receptor
FBS: fetal bovine serum
IP: intraperitoneal
K: keratin
MRU: mammary repopulating unit
NCL: nonclonogenic luminal

## References

[1] Blanpain C, Simons BD (2013). Unravelling stem cell dynamics by lineage tracing. Nat Rev Mol Cell Biol.

[2] Van Keymeulen A, Rocha AS, Ousset M, Beck B, Bouvencourt G, Rock J, Sharma N, Dekoninck S, Blanpain C (2011). Distinct stem cells contribute to mammary gland development and maintenance. Nature.

[3] de Visser KE, Ciampricotti M, Michalak EM, Tan DW, Speksnijder EN, Hau CS, Clevers H, Barker N, Jonkers J (2012). Developmental stage-specific contribution of LGR5(+) cells to basal and luminal epithelial lineages in the postnatal mammary gland. J Pathol.

[4] van Amerongen R, Bowman AN, Nusse R (2012). Developmental stage and time dictate the fate of Wnt/beta-catenin-responsive stem cells in the mammary gland. Cell Stem Cell.

[5] Feil R, Brocard J, Mascrez B, LeMeur M, Metzger D, Chambon P (1996). Ligand-activated site-specific recombination in mice. Proc Natl Acad Sci U S A.

[6] Feil R, Wagner J, Metzger D, Chambon P (1997). Regulation of Cre recombinase activity by mutated estrogen receptor ligand-binding domains. Biochem Biophys Res Commun.

[7] Indra AK, Warot X, Brocard J, Bornert JM, Xiao JH, Chambon P, Metzger D (1999). Temporally controlled site-specific mutagenesis in the basal layer of the epidermis: comparison of the recombinase activity of the tamoxifen-inducible Cre-ER(T) and Cre-ER(T2) recombinases. Nucleic Acids Res.

[8] Huh WJ, Khurana SS, Geahlen JH, Kohli K, Waller RA, Mills JC (2012). Tamoxifen induces rapid, reversible atrophy, and metaplasia in mouse stomach. Gastroenterology.

[9] Zhu Y, Huang YF, Kek C, Bulavin DV (2013). Apoptosis differently affects lineage tracing of Lgr5 and Bmi1 intestinal stem cell populations. Cell Stem Cell.

[10] Mouridsen H, Palshof T, Patterson J, Battersby L (1978). Tamoxifen in advanced breast cancer. Cancer Treat Rev.

[11] Shehata M, Teschendorff A, Sharp G, Novcic N, Russell A, Avril S, Prater M, Eirew P, Caldas C, Watson CJ, Stingl J (2012). Phenotypic and functional characterization of the luminal cell hierarchy of the mammary gland. Breast Cancer Res.

[12] Nelson JF, Felicio LS, Randall PK, Sims C, Finch CE (1982). A longitudinal study of estrous cyclicity in aging C57BL/6 J mice: I. Cycle frequency, length and vaginal cytology. Biol Reprod.

[13] Byers SL, Wiles MV, Dunn SL, Taft RA (2012). Mouse estrous cycle identification tool and images. PLoS One.

[14] Young LJT (2000). The Cleared Mammary Fat Pad and the Transplantation of Mammary Gland Morphological Structures and Cells.

[15] Hu Y, Smyth GK (2009). ELDA: extreme limiting dilution analysis for comparing depleted and enriched populations in stem cell and other assays. J Immunol Methods.

[16] Prater M, Shehata M, Watson CJ, Stingl J (2013). Enzymatic dissociation, flow cytometric analysis, and culture of normal mouse mammary tissue. Methods Mol Biol.

[17] Hovey RC, Asai-Sato M, Warri A, Terry-Koroma B, Colyn N, Ginsburg E, Vonderhaar BK (2005). Effects of neonatal exposure to diethylstilbestrol, tamoxifen, and toremifene on the BALB/c mouse mammary gland. Biol Reprod.

[18] Kotoula V, Karkavelas G, Economou L, Sionga A, Boutis L, Kerameos-Foroglou C (1993). Effects of tamoxifen and CV 205502 on the morphology and the evolution of the noncancerous mouse mammary gland. Histol Histopathol.

[19] Haslam SZ, Nummy KA (1992). The ontogeny and cellular distribution of estrogen receptors in normal mouse mammary gland. J Steroid Biochem Mol Biol.

[20] Barker N, van Oudenaarden A, Clevers H (2012). Identifying the stem cell of the intestinal crypt: strategies and pitfalls. Cell Stem Cell.

[21] Alcolea MP, Jones PH (2013). Tracking cells in their native habitat: lineage tracing in epithelial neoplasia. Nat Rev Cancer.

[22] Sherman BM, Chapler FK, Crickard K, Wycoff D (1979). Endocrine consequences of continuous antiestrogen therapy with tamoxifen in premenopausal women. J Clin Invest.

[23] Jordan VC, Fritz NF, Langan-Fahey S, Thompson M, Tormey DC (1991). Alteration of endocrine parameters in premenopausal women with breast cancer during long-term adjuvant therapy with tamoxifen as the single agent. J Natl Cancer Inst.

[24] Groom GV, Griffiths K (1976). Effect of the anti-oestrogen tamoxifen on plasma levels of luteinizing hormone, follicle-stimulating hormone, prolactin, oestradiol and progesterone in normal pre-menopausal women. J Endocrinol.

[25] Rios AC, Fu NY, Lindeman GJ, Visvader JE (2014). In situ identification of bipotent stem cells in the mammary gland. Nature.

[26] Osborne CK, Boldt DH, Clark GM, Trent JM (1983). Effects of tamoxifen on human breast cancer cell cycle kinetics: accumulation of cells in early G1 phase. Cancer Res.

[27] Taylor IW, Hodson PJ, Green MD, Sutherland RL (1983). Effects of tamoxifen on cell cycle progression of synchronous MCF-7 human mammary carcinoma cells. Cancer Res.

[28] Robinson SP, Langan-Fahey SM, Johnson DA, Jordan VC (1991). Metabolites, pharmacodynamics, and pharmacokinetics of tamoxifen in rats and mice compared to the breast cancer patient. Drug Metab Dispos.

[29] Fong CJ, Burgoon LD, Williams KJ, Forgacs AL, Zacharewski TR (2007). Comparative temporal and dose-dependent morphological and transcriptional uterine effects elicited by tamoxifen and ethynylestradiol in immature, ovariectomized mice. BMC Genomics.

[30] Williams C, Edvardsson K, Lewandowski SA, Strom A, Gustafsson JA (2008). A genome-wide study of the repressive effects of estrogen receptor beta on estrogen receptor alpha signaling in breast cancer cells. Oncogene.

[31] Mandlekar S, Kong AN (2001). Mechanisms of tamoxifen-induced apoptosis. Apoptosis.

[32] Mandlekar S, Yu R, Tan TH, Kong AN (2000). Activation of caspase-3 and c-Jun NH2-terminal kinase-1 signaling pathways in tamoxifen-induced apoptosis of human breast cancer cells. Cancer Res.

[33] Beleut M, Rajaram RD, Caikovski M, Ayyanan A, Germano D, Choi Y, Schneider P, Brisken C (2010). Two distinct mechanisms underlie progesterone-induced proliferation in the mammary gland. Proc Natl Acad Sci U S A.

