# Supplementary material for: The influence of tamoxifen on normal mouse mammary gland homeostasis
Source: Breast Cancer Res. 2014 Jul 24;16:411. doi: 10.1186/s13058-014-0411-0 (PMC4303226; doi:10.1186/s13058-014-0411-0)
Supplement: Supplementary file 1 — Additional file 1: Table S1.: Summary of lineage-tracing studies in the mammary epithelium. (PDF 8 KB) [file 13058_2014_411_MOESM1_ESM.pdf]

Table S1: Summary of lineage-tracing studies in the mammary epithelium

Reference	System used	Total dose TAM received	Age of treatment
Mammary gland			
Van Keymeulen et al, 2011, Nature	K18-CreER<br>K8-CreER<br>K5-CreER<br>Lgr5-CreER^{T2}	3x5 mg/mouse<br>3x5 mg/mouse<br>3x5 mg/mouse<br>3x5 mg/mouse	P28, P56<br>P28, P56<br>P28, P56<br>P28, P56
Van Amerongen et al., 2012, CSC	Axin2-CreER^{T2}	1.5 mg/mouse<br>2.5 mg/mouse<br>4 mg/mouse	P14<br>P28<br>P56
De Visser et al, 2012, J Path.	Lgr5-CreER^{T2}<br><br>R26-CreER^{T2}	0.5 mg/mouse<br>1.5 mg/mouse<br>0.5 mg/mouse<br>1.5 mg/mouse	P1<br>P12<br>P1<br>P12
Skin			
Jaks et al., 2008, Nature Genetics	Lgr5-CreER^{T2}	0.5 mg/mouse	P21
Snippert et al., 2010, Science	Lgr6-CreER^{T2}	2 mg/mouse	Adult
Intestine			
Barker et al., 2007, Nature	Lgr5-CreER^{T2}	2 mg/mouse	Adult
Yan et al., 2012, PNAS	Lgr5-CreER^{T2}<br>Bmi1-CreER	5 mg/mouse<br>5 mg/mouse	Adult<br>Adult
