# Supplementary figures and images for: The influence of tamoxifen on normal mouse mammary gland homeostasis
Source: Breast Cancer Res. 2014 Jul 24;16:411. doi: 10.1186/s13058-014-0411-0 (PMC4303226; doi:10.1186/s13058-014-0411-0)

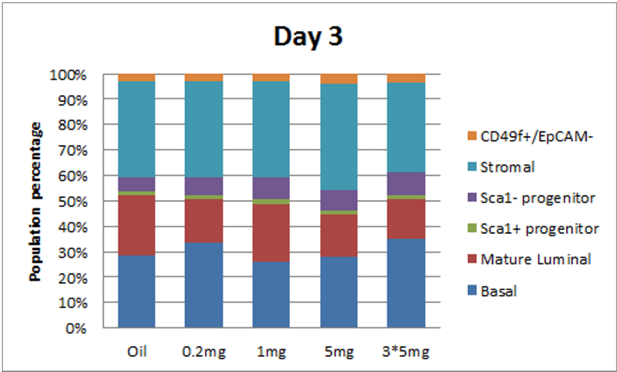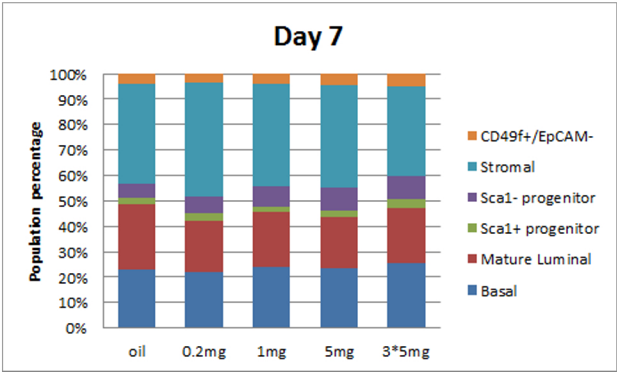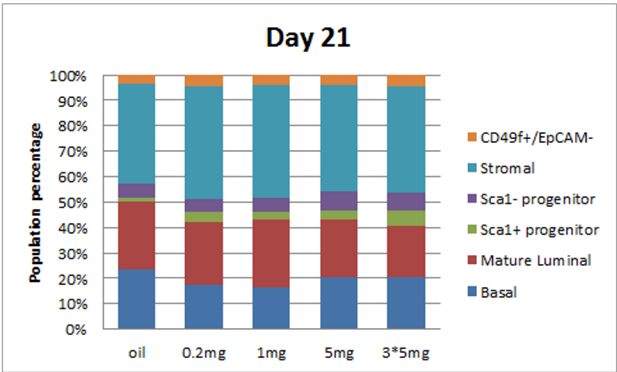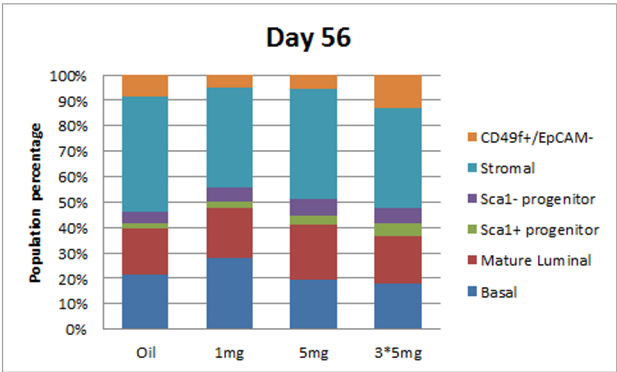

Supplement: Supplementary file 2 — Additional file 2: Figure S1.: Distribution of mammary cell types in mice injected with oil or with varying doses of tamoxifen and analyzed 3 to 56 days later. (PDF 2 MB) [file 13058_2014_411_MOESM2_ESM.pdf]

Day 1

Day 2

Day 3

Oil

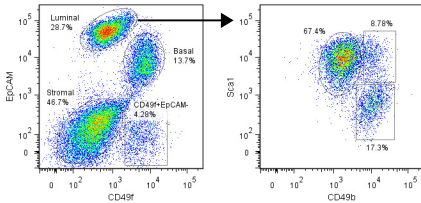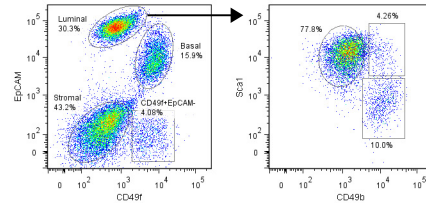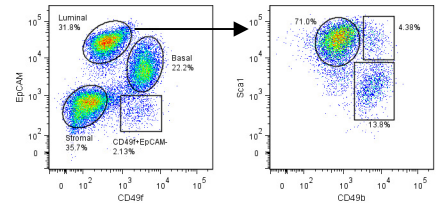

1 mg

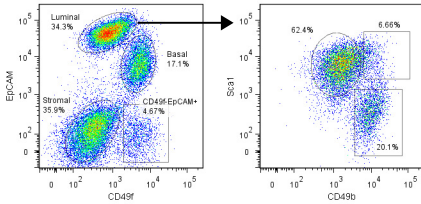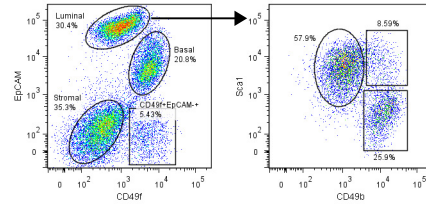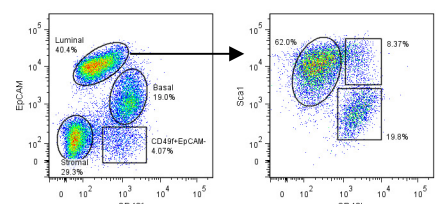

5 mg

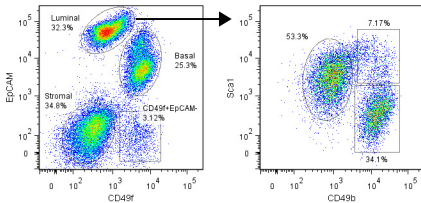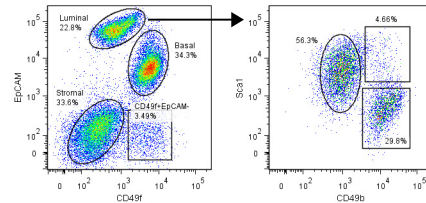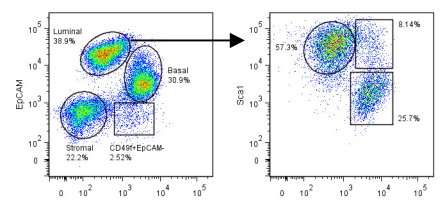

3\*5 mg

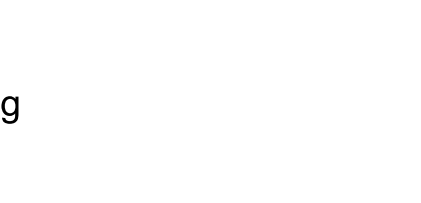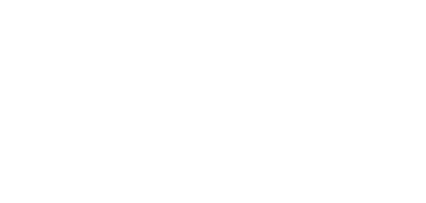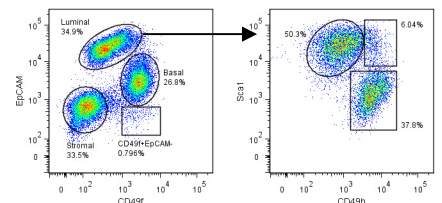

Day 7

Day 21

Day 56

Oil

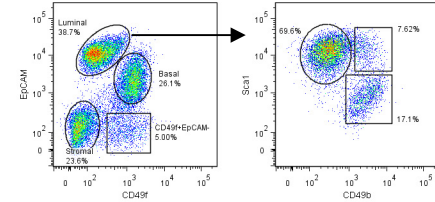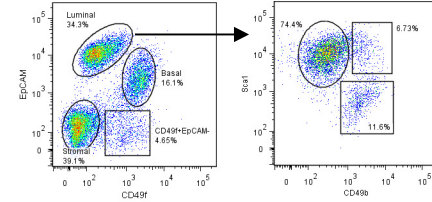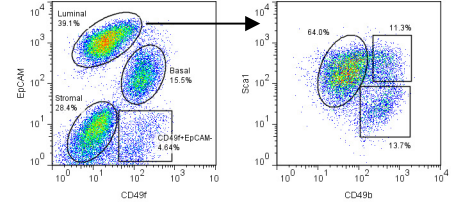

1 mg

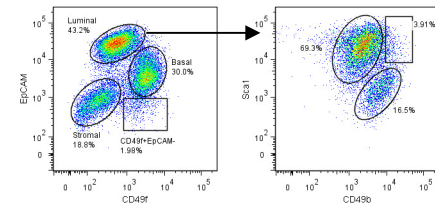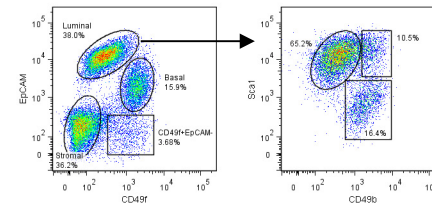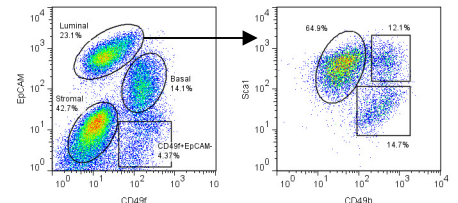

5 mg

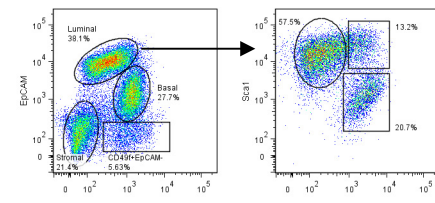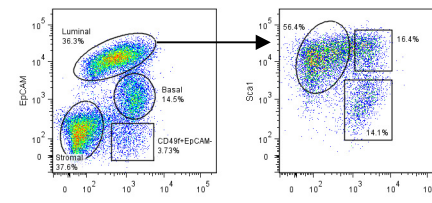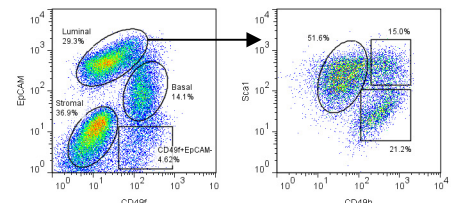

3\*5 mg

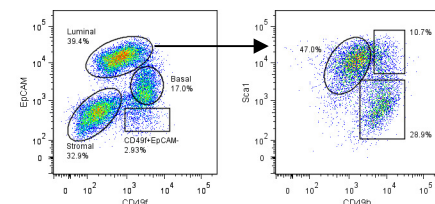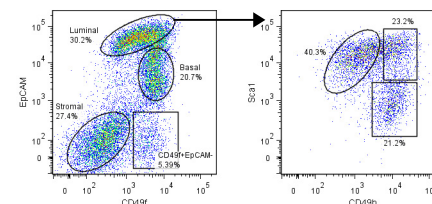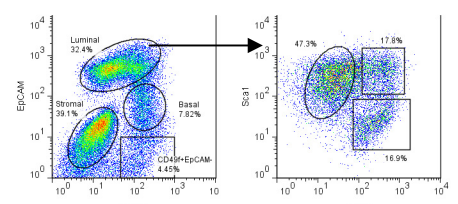

Supplement: Supplementary file 3 — Additional file 3: Figure S2.: Gating of mouse mammary epithelial and luminal subpopulations of different doses analyzed at various time points. (PDF 3 MB) [file 13058_2014_411_MOESM3_ESM.pdf]

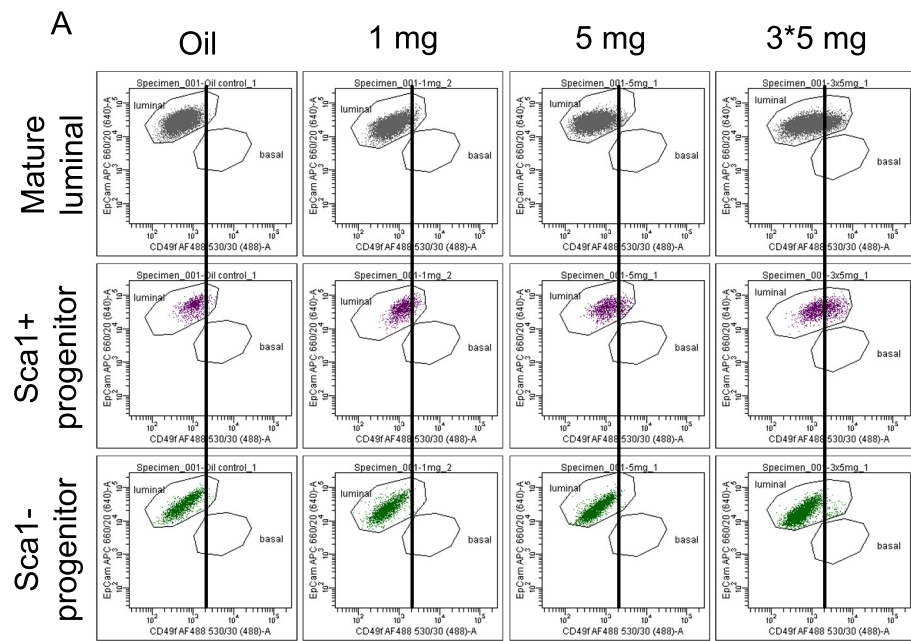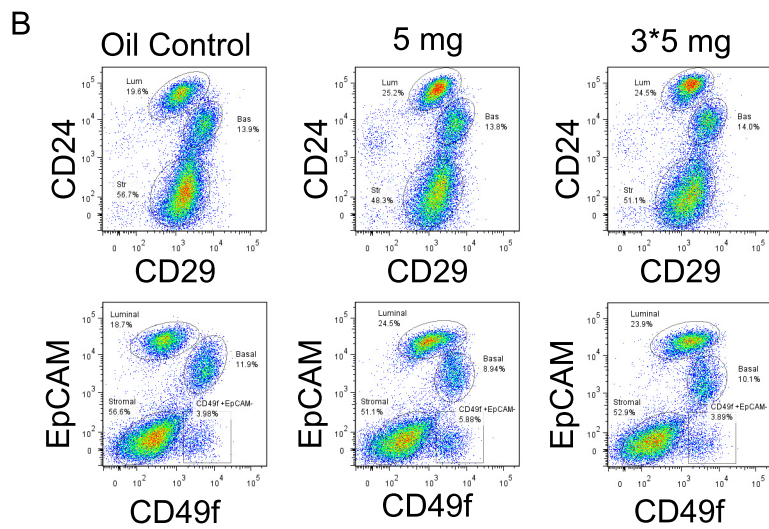

Supplement: Supplementary file 4 — Additional file 4: Figure S3.: (A) Back-gating of NCL, Sca1+, and Sca1− progenitors from various doses analyzed 21 days after tamoxifen administration. Solid black line, CD49f expression levels in oil-treated control mice. (B) Comparing mouse epithelial subpopulations with CD24/CD29 or EpCAM/CD49f at Day 21. (PDF 1 MB) [file 13058_2014_411_MOESM4_ESM.pdf]

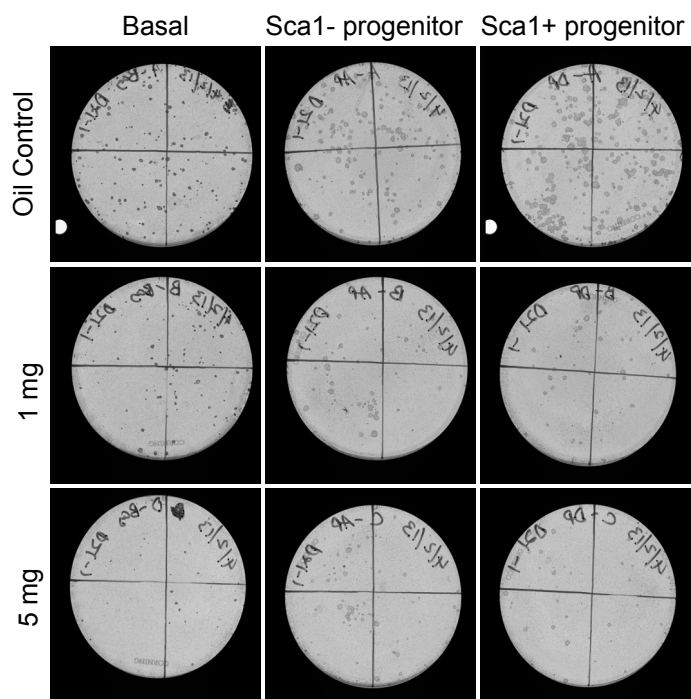

Supplement: Supplementary file 5 — Additional file 5: Figure S4.: CFC plates from a representative experiment 21 days after treatment. (PDF 460 KB) [file 13058_2014_411_MOESM5_ESM.pdf]

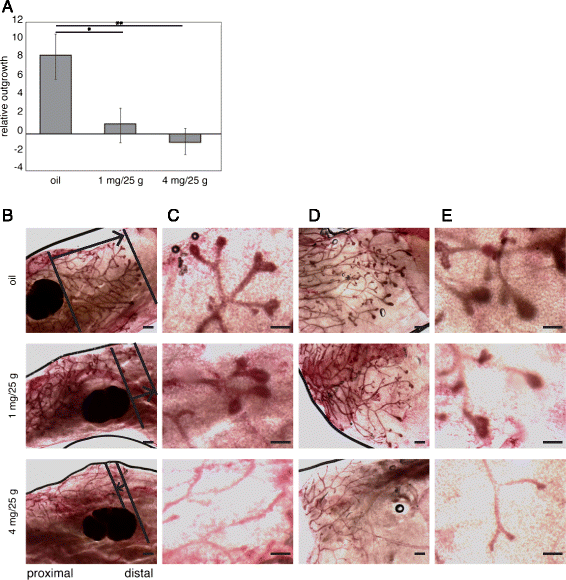

Supplement: Supplementary file 8 — Authors’ original file for figure 1 [file 13058_2014_411_MOESM8_ESM.gif]

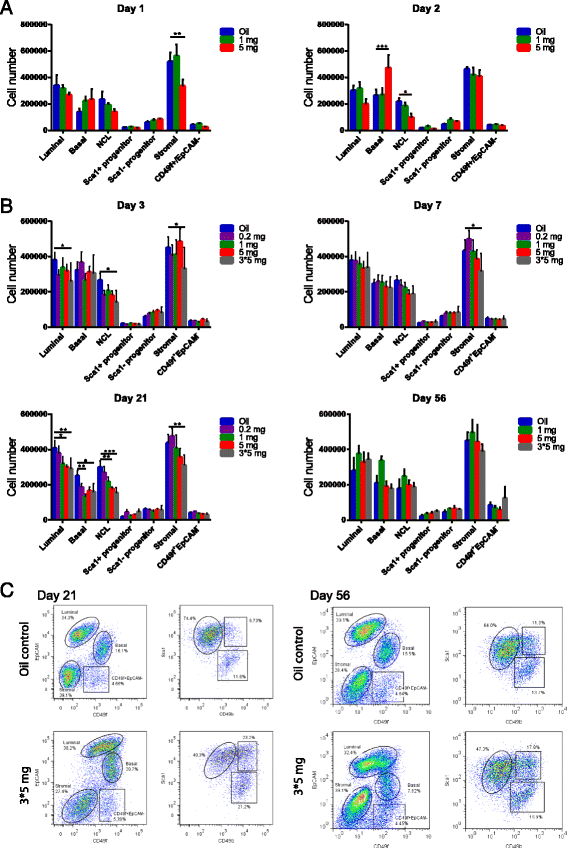

Supplement: Supplementary file 9 — Authors’ original file for figure 2 [file 13058_2014_411_MOESM9_ESM.gif]

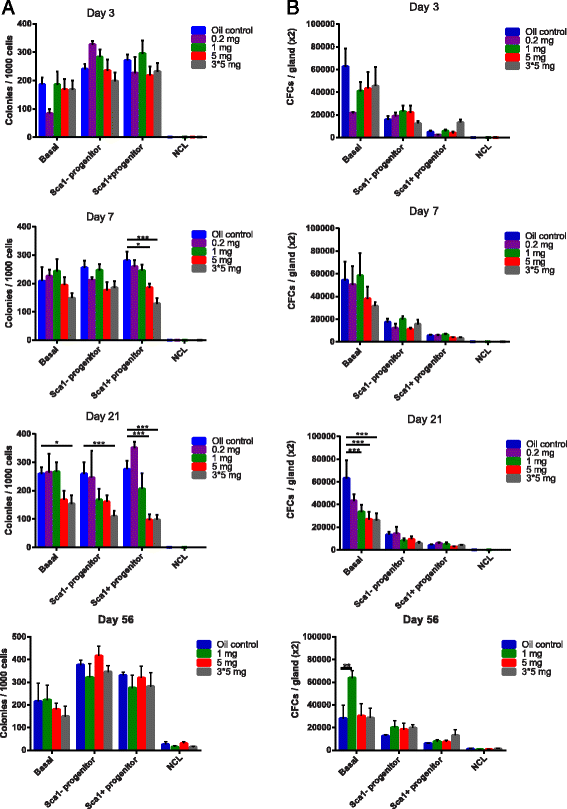

Supplement: Supplementary file 10 — Authors’ original file for figure 3 [file 13058_2014_411_MOESM10_ESM.gif]

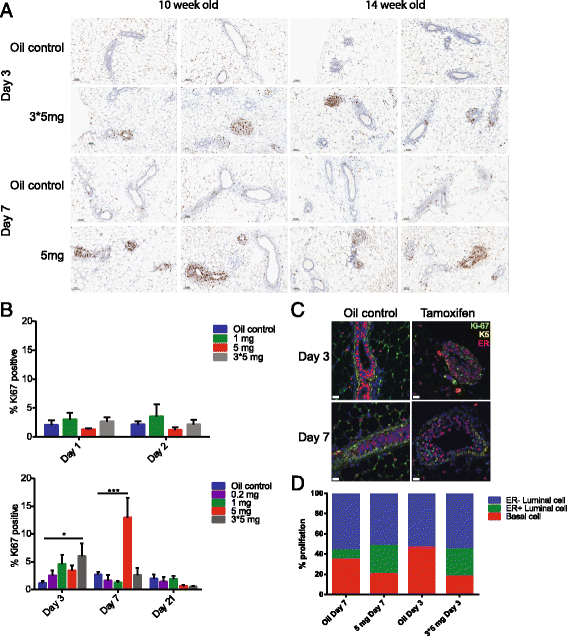

Supplement: Supplementary file 11 — Authors’ original file for figure 4 [file 13058_2014_411_MOESM11_ESM.gif]

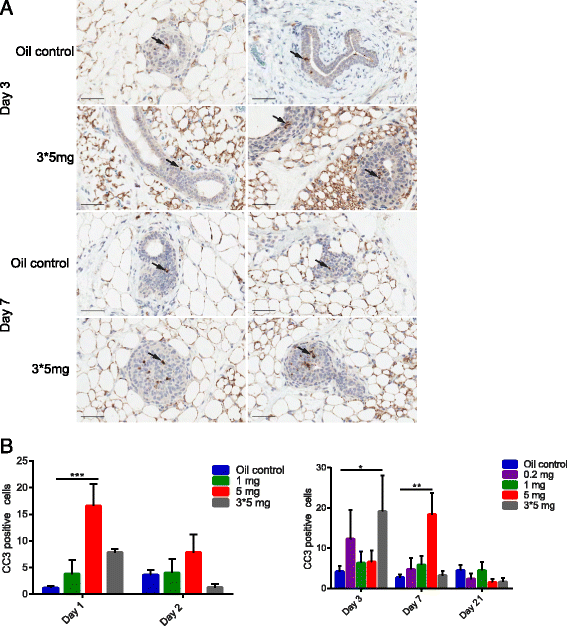

Supplement: Supplementary file 12 — Authors’ original file for figure 5 [file 13058_2014_411_MOESM12_ESM.gif]
