# Supplementary material for: The influence of tamoxifen on normal mouse mammary gland homeostasis
Source: Breast Cancer Res. 2014 Jul 24;16:411. doi: 10.1186/s13058-014-0411-0 (PMC4303226; doi:10.1186/s13058-014-0411-0)
Supplement: Supplementary file 6 — Additional file 6: Table S2.: Frequency and absolute number of MRUs in oil- and tamoxifen-treated mice. (PDF 13 KB) [file 13058_2014_411_MOESM6_ESM.pdf]

Table S2: Frequency and absolute number of MRUs in oil- and tamoxifen- treated mice.

Dose of tam	Cell dose	Take rate		% fat pad filled		MRU frequency (95% CI)				MRUs/pair of glands	
		Day 3	Day 21	Day 3	Day 21	Day 3		Day 21		Day 3	Day 21
Oil control	750	5/8	8/10	100, 100, 100, 75-100, <25	75-100, 25-50, 75-100, 50, 50-75, 100,100, 100	1/765 (1/1,904-1/308)		1/467 (1/1,007-1/216)		3,399	5,139
1 mg	750	2/6	9/10	100, 100	25-50, 50, 100, 100, 100, 75-100, 50, 75-100, 100	1/1,850 (1/7,467-1/458)	ND	1/326 (1/731-1/146)	ND	1,155	6,173
5 mg	750	7/8	8/10	75-100, 50, 100, 75-100, 75-100, 25-50, 75-100	100, <25, 50, 50-75, 75-100, <25, 100, 50	1/361 (1/871-1/149)		1/467 (1/1,007-1/216)		4,467	2,489

ND = No significant difference
