# Supplementary material for: The influence of tamoxifen on normal mouse mammary gland homeostasis
Source: Breast Cancer Res. 2014 Jul 24;16:411. doi: 10.1186/s13058-014-0411-0 (PMC4303226; doi:10.1186/s13058-014-0411-0)
Supplement: Supplementary file 7 — Additional file 7: Table S3.: Distribution of mice in different stages of the estrus cycle after tamoxifen treatment. (PDF 13 KB) [file 13058_2014_411_MOESM7_ESM.pdf]

Table S3 : Distribution of mice in different stages of the estrus cycle post-tamoxifen treatment.

Time point	Dose	Estrus stage	
Day 1	Oil<br>1 mg<br>5 mg	P, P, E, M, D<br>E, E, P/E, M, M<br>E, P, D, M, D	
Day 2	Oil<br>1 mg<br>5 mg	M, E, D, E, D<br>E, P/E, M, E, E<br>E, P, E, M, E	
Day 3	Oil<br>0.2 mg<br>1 mg<br>5 mg<br>3*5 mg	E, E/M, P, E/M, M, M, M, D, M<br>E, E/M, E, E/M, E, M, E/M<br>E, M, E, E, P, E, E, M, P<br>E, E, E, E/M, E/M, E/M, E, P, M, M<br>P, M, M, M, M, P, M, M, M, M, M	P = 0.060
Day 7	Oil<br>0.2 mg<br>1 mg<br>5 mg<br>3*5 mg	M, D, E, E, E, M, D, D, E<br>M, D, M, P, M, M, D<br>M, P/E, M, E, M, M/D, M, P, M<br>M, M, M, M, M, M, E/M, M, M, M, M<br>P, P/E, P/E, E, E/M, E/M, M, M, M	P = 0.070
Day 21	Oil<br>0.2 mg<br>1 mg<br>5 mg<br>3*5 mg	M, M, E, D, D, P, M, D, P<br>E/M, E, E, D, M, D<br>E, M, P, P, P, E, P, M, E<br>P, D, E, E, M, M, D, D, E, M<br>M, E/M, D, D, M, M, M, M, M, M, M	
